# But I Trust My Teen: Parents' Attitudes and Response to a Parental Monitoring Intervention

**DOI:** 10.1155/2012/396163

**Published:** 2012-06-07

**Authors:** Aaron Metzger, Christa Ice, Lesley Cottrell

**Affiliations:** ^1^Department of Psychology, West Virginia University, 1124 Life Sciences Building, Morgantown, WV 26506-6040, USA; ^2^Department of Biostatistics, West Virginia University School of Public Health, P.O. Box 9190, Morgantown, WV 26506-9190, USA; ^3^Department of Pediatrics, RCBHSC, West Virginia University School of Medicine, P.O. Box 9214, Morgantown, WV 26506-9214, USA

## Abstract

Parental knowledge gained from monitoring activities protects against adolescent risk involvement. Parental monitoring approaches are varied and may be modified with successful interventions but not all parents or adolescents respond to monitoring programs the same way. 339 parent-adolescent dyads randomized to receive a parental monitoring intervention and 169 parent-adolescent dyads in the control group were followed for one year over four measurement periods. Parent attitudes about the usefulness of monitoring, the importance of trust and respecting their teens' privacy, and the appropriateness of adolescent risk-taking behavior and experimentation were examined as predictors of longitudinal change in parental monitoring and open communication. Similar effects were found in both the intervention and control group models regarding open communication. Parental attitudes impacted longitudinal patterns of teen-reported parent monitoring, and these patterns differed across experimental groups. In the intervention group, parents' beliefs about the importance of trust and privacy were associated with a steeper decline in monitoring across time. Finally, parents' attitudes about the normative nature of teen experimentation were associated with a quadratic parental monitoring time trend in the intervention but not the control group. These findings suggest that parental attitudes may impact how families respond to an adolescent risk intervention.

## 1. Introduction

 Adolescent risk behaviors such as drinking alcohol, other drug use, and early unprotected sexual activity often cooccur in a cluster of health risk behaviors that can lead to heightened risks of adolescent pregnancy, sexually transmitted diseases (STDs), and the human immunodeficiency virus (HIV) [1]. Behavioral interventions for adolescents have established a strong foundation noting improved protection by improving adolescent HIV/AIDS and protective behavior knowledge, attitudes, and skills [2–5]. Despite successful adolescent risk reduction programming, HIV infections of young persons (13–29 years old) in the United States continue to impact 39% of all new HIV infections in 2009 [6]. Finding diverse and effective ways to educate and motivate adolescents to reduce adolescent risk behavior continues to be a challenge.

 The extent to which parents know their adolescents' activities, friends, and whereabouts (i.e., parental monitoring knowledge) has a documented influence on adolescent risk involvement [7]. Parental monitoring is most successful when the information comes directly from the adolescent. Therefore, open communication between parents and adolescents is an essential means for improving parental monitoring knowledge [8, 9]. However, not all monitoring strategies are direct [10, 11]. Parents might also use indirect methods for obtaining information such as asking friends or reading emails and journals [10].

 The parental monitoring process changes over time with the age of the adolescent, the nature of the parent-adolescent relationship, and any events that may have been acknowledged [12]. Parents and adolescents perceive parents' monitoring efforts differently [13]. Both groups come into the relationship with different attitudes about what monitoring should entail, when it should occur, and how effective it is on a regular basis. These factors may change over time as adolescents seek greater independence and freedoms [7]. Parental monitoring strategies, knowledge, and attitudes also differ by adolescent age and gender, parent characteristics such as income and work schedule, and the home environment (e.g., number of children in the home, number of adults) [7, 14, 15]. For example, parents monitor their younger children and daughters more than their older children and sons. Less monitoring is noted in homes of single parents, multiple children, or among low-income families [7].

 The balance between employing effective monitoring strategies while respecting adolescents' privacy and building trust is a common concern for parents and a phenomenon that has been studied more closely over the past decade [12]. Strategies such as negotiated unsupervised time have mixed results in terms of adolescent experimentation. However, the process of the negotiations involving parent-adolescent communication, regardless of the level of experimentation, successfully builds parent trust and a feeling of respect for the adolescents which serves as a protective factor for many risk behaviors among males and females [16].

 Efforts to improve parent-adolescent communication and the parental monitoring process have been made to reduce adolescent risk behavior and, in turn, reduce adolescents' risks of subsequent health problems. Stanton and colleagues [17] developed and evaluated a parental monitoring program for inner city families. The successful home delivery of the Informed Parents and Children Together (ImPACT) program resulted in improvements in the agreement between parents and adolescents about adolescents' involvement in select risk (or protective) behaviors. Other programs have documented similar success improving parent and adolescent communication and increasing parental monitoring knowledge [18, 19]. Despite this success, all families enrolled in these and other programs are not equally impacted by a given curriculum. Given that these programs have not been successful for all families, there is a critical need to identify specific characteristics that contribute to a lack of response to a given intervention.

 In the present study, we examined parent-adolescent dyads who participated in a program designed to improve parent-adolescent communication, parental monitoring knowledge, and parental monitoring strategies. Overall, the program improved adolescent-reported parental monitoring knowledge of their parents. Parental monitoring knowledge was associated with limited or reduced adolescent risk behaviors. However, like other parental monitoring programs, not all parents and adolescents responded to the intervention in the desired way. The interaction between parent and adolescent was an important element of the intervention. Having one individual not responding to the intervention would be potentially detrimental to the impact of the program for the dyad as a whole. We hypothesized that this outcome was partially due to the variability in parents' attitudes about monitoring.

 Prior to the start of an intervention, parents have established attitudes about the extent to which a certain type and amount of monitoring protects their adolescents from risky behaviors [20]. Parents may also have strong attitudes about the level they prefer to trust and respect the privacy of their adolescents [21, 22]. Finally, parents may pair a certain level of monitoring with their existing attitudes about the extent to which adolescents will naturally experiment with risky behaviors. These attitudes may not only influence adolescent risk behaviors but also strongly influence parents' monitoring and reaction to interventions as a result.

 We might expect that these preintervention attitudes may impact the level to which parents invest in an intervention and also the degree to which their actual behaviors would change after the intervention. This parental monitoring program was also family based requiring both parents and adolescents to communicate about monitoring and risk behaviors. This study, therefore, provides an additional opportunity to examine how preintervention attitudinal factors impact both parent and adolescent reports of communication and monitoring over time.

## 2. Materials and Methods

### 2.1. Sample Recruitment

 Adolescents (12–17 years) enrolled in high schools throughout 15 counties of a rural, eastern state were eligible to participate in the parental monitoring program. School administrators provided initial approval to distribute information about the study to eligible students. The informational letter described the purpose and procedures of the study and included the appropriate consent/assent documents. Participation required parent consent and participation in the study.

### 2.2. Measures

#### 2.2.1. Parent Reported Attitudes about Adolescent Risk Behavior

 Three subscales were used from the parental monitoring attitudes scale, including parent attitudes about (1) the usefulness of monitoring; (2) the impact of monitoring on adolescent risk behavior and experimentation; (3) the importance of respecting adolescent trust and privacy through one's monitoring efforts. All scales were on a four point Likert response scale; responses ranged from disagree very strongly to agree very strongly. Reliability was assessed for the baseline responses.

 Parent attitudes about the usefulness of the monitoring process (UMP) included 11 items; items were averaged for a scale score. Higher scores indicated stronger agreement. The internal reliability of this scale was 0.81. Example item, “it is important for parents to ask for details when children are spending time with their friends outside of the home.”

 Parent attitudes about the impact of monitoring on adolescent risk behavior and experimentation (EXP) included 11 items; items were averaged for a scale score. Higher scores indicated stronger agreement. Internal reliability for the EXP subscale was 0.77. Example item, “as kids get older, they will experiment with alcohol and drugs.”

 Parent attitudes about monitoring and the importance of adolescent trust and privacy (ATP) included 4 items; items were averaged for a scale score. Higher scores indicated stronger agreement. Internal reliability for the ATP subscale was 0.84. Example item “trusting kids means not checking on them while they are out.”

#### 2.2.2. Parent-Adolescent Open Communication

 The open communication subscale of the Parent-Adolescent Communication Scale [23] includes 10 items such as “my child can discuss beliefs without feeling restrained or embarrassed.” Parents and adolescents completed modified versions of the scale, only parent reports are used in these analyses. Responses ranged from 1 being “strongly disagree” to 4 being “strongly agree”. Items were averaged for a scale score. Higher scores indicated greater agreement for having an open communication process with the parent (or adolescent). The open communication subscale based on parent report had a strong internal reliability (Cronbach's alpha = 0.87) at baseline.

#### 2.2.3. Parent Monitoring Strategies

 The parental monitoring instrument (PMI) was used to assess parents' monitoring strategies [24]. The PMI is composed of three major subscales representing different monitoring strategies: direct, indirect, and restrictive. Parents and adolescents completed the PMI at each assessment point. Only adolescent-reported direct monitoring strategies were examined in this study.

 Direct monitoring strategies represent monitoring that involves direct communication between the parent and adolescent. Items such as “in the past 4 months, how many times has your parent done the following: asked you about specifics of planned activities before giving permission to attend?” were answered using a 4-point Likert scale ranging from 1 “0 times” to 4 “5 + times.” Three items were averaged to obtain the scale score; higher scores indicated more frequent use of direct monitoring. The internal reliability for the direct monitoring strategy based on adolescent report at baseline was strong (Cronbach's alpha = 0.84).

### 2.3. Procedures

 Once consent and assent was obtained, each parent-adolescent dyad received two separate questionnaire packets (one for adolescent; one for parent). Separate self-addressed and stamped envelopes were provided to return the completed questionnaires. Each adolescent received $15 and each parent received $15 for completing their respective questionnaires.

 Parent-adolescent dyads were randomized to one of two groups at baseline. The intervention group received a parental monitoring program, Communication Between Parents and Adolescents* (COPA)*. The COPA Program was DVD based and could be reviewed within the home. The parent and adolescent were required to watch the DVD together and to respond to workbook questions as they progressed through the DVD. The COPA DVD was divided into four video segments each representing a scenario in which an adolescent may engage in risky behaviors. Within two weeks of receiving the DVD in the mail, parents and adolescents discussed the scenarios and responded to additional questions about what they would do to respond if in a similar situation.

 Dyads randomized to the control group received an interactive DVD that was organized into the same number of segments and included similar workbook activities as the intervention. The focus of the control curriculum was on family roles and recycling. Workbook questions related to how family members work together to improve recycling within the home. No discussions of monitoring or communication about risk were incorporated into the workbook or scenario building.

 Parents completed a series of questionnaires related to parental monitoring strategies, monitoring knowledge, communication about risk behaviors, monitoring attitudes, and adolescent risk involvement at baseline and at three points after-intervention (4, 8, and 12 months). At each assessment point, separate questionnaires were mailed to participating parents and adolescents with a separate envelope for return. For the purposes of this study, parent and adolescent reports of parents' monitoring attitudes and the level of open communication within their relationship was used. Study procedures were approved by the West Virginia University Institutional Review Board.

### 2.4. Analyses

 The present study utilized multilevel hierarchical linear growth modeling to examine parental monitoring attitude predictors of baseline differences and change over time in open communication and parents' use of direct monitoring strategies among parent-adolescent dyads, separated by experimental group. All continuous variables were examined for normality assumptions. Data was organized in IBM SPSS 20.0 and analyzed in SAS 9.2 and SSI's HLM 7.0. Multilevel analysis was used because it can account for clustering of data within individuals and is robust against problems arising from issues of unequal cell sizes and independence of errors. Mixed effect models also can accommodate missing data points (individuals who miss one or multiple sessions), an especially important characteristic given the attrition levels across the four waves of the study. Linear and quadratic effects were examined, as the intervention occurred between the first and second time point of the year-long assessment.

 Four separate growth models were examined. First, models were run within the intervention sample only: one for parent-reported open communication and one for teen-reported parental monitoring behaviors. In each of the models, time (4 waves) was nested within family. An initial model was run to examine the amount of clustering in the data as captured in the intraclass correlation (ICC). Next, a growth model was run with both linear and quadratic trend coefficients to examine the total amount of variance in intercepts and time slopes. Finally, family demographic characteristics (adolescent age, adolescent gender, parent gender, and family income) and parent-reported attitudes concerning adolescent behavior and monitoring (EXP, UMP, ATP) were included as predictors of the intercepts (baseline differences) and slopes (time × predictor interaction) for each model. Parental attitude variables were grand-centered around the mean. Variables that were not associated with the intercepts and slopes (*P* > .10) were trimmed from each model.

 In order to determine whether the same pattern of predictors from the intervention models was also predictive in the control group, two separate growth models were run within the control sample for parent-reported open communication and teen-reported parental monitoring. The same variables from the intervention group models were included as predictors of intercepts and slopes. Individual parameters could then be compared across intervention and control group models.

## 3. Results

### 3.1. Sample Characteristics

 Characteristics of the parent-adolescent dyads who participated in the intervention and control are provided in Table 1. Across group, the majority of adolescents participating in the study were female and Caucasian. The ethnicity of this sample mirrors the ethnic distribution of the rural area from which the sample was enrolled. The mean adolescent age of the intervention group was 14.5 years old (12–17 years); the mean parent age was 37.2 years. The mean adolescent age of the control group was slightly older at 15 years old (12–18 years); the mean parent age was also older at 41 years. The majority of families who participated had two adults in the home and two children. Slightly less than 20 percent of families were living at or below the poverty line for both control and intervention groups. Descriptive statistics for each of the study variables over time are also provided in Table 1. On average, most parents openly communicated with their adolescents on a regular basis over time. Adolescents, on average, reported that their parents sometimes used the direct monitoring strategies at baseline, 4, 8, and 12 months after intervention.

 Pearson correlation associations among all study variables for the intervention group are presented in Table 2. Parent attitudes about the usefulness of monitoring were associated with attitudes about trust and privacy (*P* < .001) and adolescent experimentation (*P* < .001). Parent attitudes about the usefulness of monitoring were also associated with parent-reported open communication at baseline and all postintervention assessment points (*P* < .001). Parent attitudes about adolescent experimentation were associated with parent open communication at baseline (*P* < .001), 4 months (*P* < .001), and 12 months after intervention (*P* < .001). Parent attitudes about trust and privacy issues were not significantly associated with parent-reported open communication except at baseline (*P* < .001). Adolescent reports of parents' direct monitoring were not significantly associated with parent reports of open communication. The only parental attitudes associated with adolescent report of direct monitoring were those related to adolescent trust and privacy (*P* < .001).

 In the results below, we first present the models found within the intervention group for both parent open communication and parent direct monitoring. We then present findings of applying the intervention models to the control group.

### 3.2. Parent Open Communication (Intervention Group)

 An initial linear growth model of the intervention group indicated that parents' self-reported open communication scores were significantly clustered within individuals (ICC = .58). In addition, there was a significant amount of interindividual variance in open communication intercepts (variance = .096, *χ* ^2^ = 691.21,*P* < .001) and linear slopes (variance = .004, *χ* ^2^ = 266.87, *P* < .001). There were no significant amounts of variance in the quadratic term, so it was trimmed from the model. The final trimmed model accounted for 47% of the variance in intercepts and 22% of the variance in linear slopes. At baseline, mothers enrolled in the intervention group reported significantly higher levels of open communication than fathers in the intervention group (Table 3). Parents of older children reported marginally higher levels of open communication than parents of younger children, while increasing family income was associated with lower levels of open communication at baseline. Two parental attitudes were associated with parent-reported open communication at baseline. Parents who strongly endorsed monitoring for protective means viewed their relationship with their adolescents as being more open at baseline than other parents. Parents who believed adolescent risk-taking and experimentation were normative perceived their relationships with their adolescents as being less open in terms of their communication than other parents at baseline.

**Figure 1:**
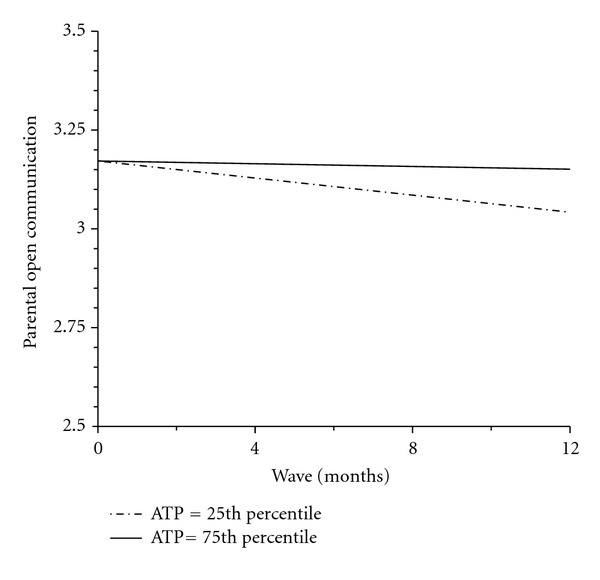
Parental open communication slopes at different levels of ATP. ATP: parent attitudes about monitoring and the importance of adolescent trust and privacy.

 Parental beliefs concerning trust in teens and respect for teens' privacy were associated with change in open communication levels over time. Parents who strongly believed trusting their adolescents meant not touching base with them or asking for information from their adolescents reported stable levels of open communication (75th percentile; Figure 1). In contrast, parents who expressed lower levels of trust and respect for their teens' privacy reported a decline in open communication after intervention (25th percentile; Figure 1).

### 3.3. Teen-Reported Parental Monitoring Behavior (Intervention Group)

 An initial model of the intervention group indicated that teen-reported parental monitoring scores were significantly clustered within individuals (ICC = .59). In addition, the model indicated significant amounts of interindividual variance in monitoring intercepts (variance = .613, *χ* ^2^ = 584.96, *P* < .001), linear slopes (variance = .223, *χ* ^2^ = 230.79, *P* = .014), and quadratic slopes (variance = .023, *χ* ^2^ = 232.37, *P* = .012). The final trimmed model accounted for 11% of the variance in intercepts, 14% of the variance in linear slopes, and 12% of the variance in quadratic slopes. At baseline, females and adolescents from families with higher family incomes reported higher levels of parental monitoring (Table 4).

**Figure 2:**
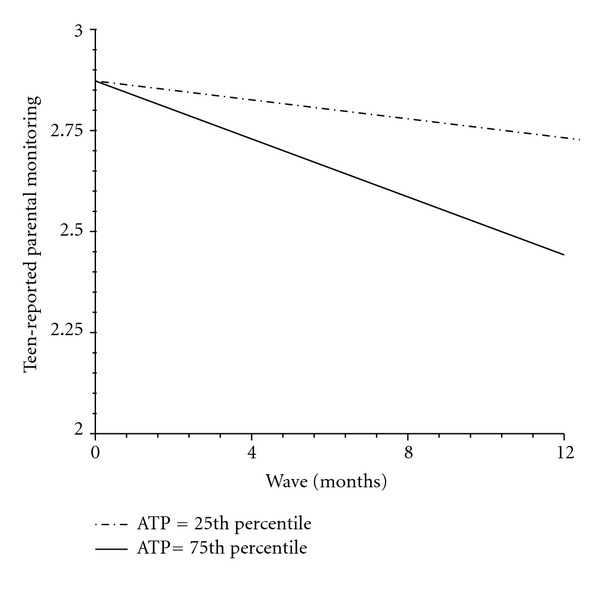
Teen-reported parental monitoring slopes at different levels of ATP. ATP: parent attitudes about monitoring and the importance of adolescent trust and privacy.

**Figure 3:**
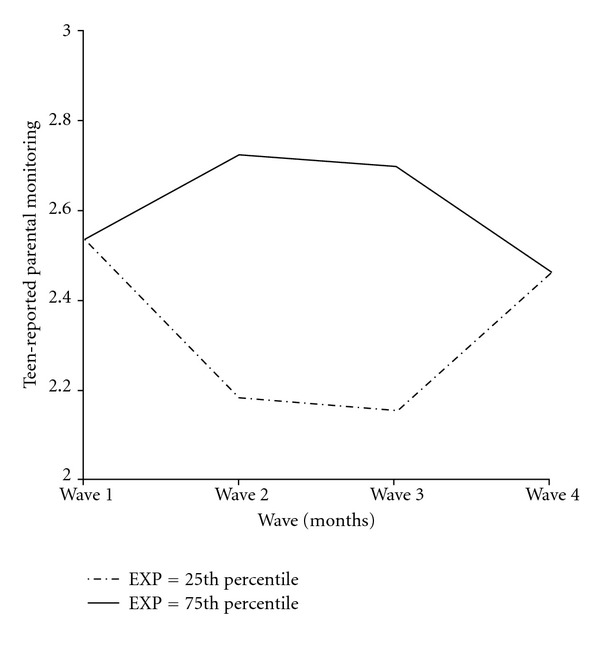
Teen-reported parental monitoring linear + quadratic slopes at different levels of EXP. EXP: parent attitudes about the impact of monitoring on adolescent risk behavior and experimentation.

 Teen-reported parental monitoring decreased marginally across the four waves for the entire intervention group, but this trend was exacerbated by parents' attitudes about trust and privacy. Specifically, adolescents of parents who expressed higher levels of respect for teens' privacy and trust in teens reported a greater decline in their parents' direct monitoring over time than adolescents in the intervention group whose parents strongly disagreed to limiting monitoring to safeguard adolescent trust and privacy (Figure 2). In addition, parents' baseline attitudes toward adolescent experimentation (i.e., adolescents will do it regardless of what parent does) accounted for individual differences in both linear and quadratic slopes (Figure 3). Specifically, when parents did not view teen risk behavior as something to be expected, adolescent perceived reports of direct monitoring initially declined but then increased across the remaining waves. The opposite pattern emerged for adolescents whose parents more strongly believed that adolescent experimentation with risk behavior is normative. For these adolescents, direct parental monitoring initially increased after the intervention, but then gradually decreased across the remaining measurement waves.

### 3.4. Control Group: Comparison Models

 Follow-up analyses were run to explore whether similar associations between parenting beliefs about monitoring and both parent open communication and teen-reported monitoring were evident in the control group (*n* = 169). Identical models were tested on the control group, which contained the same variables from the final trimmed models described above. Findings are reported in Tables 3 and 4.

#### 3.4.1. Open Communication

Although there were several differences in demographic predictors of intercepts, compared to the intervention group model, the model for parent-reported open communication within the control group indicated nearly identical patterns of associations between parenting beliefs and open communication intercepts and slopes. Specifically, parents' positive endorsement of monitoring behaviors was associated with greater open communication at baseline, and parents' beliefs that adolescent risk behavior and experimentation was normative were associated with lower levels of open communication at baseline. In addition, and also similar to the intervention group model, parents' attitudes toward teen trust and privacy were positively associated with linear change in parent-reported open communication across the four waves of the study. Thus, parenting beliefs were associated with parent-reported open communication intercepts and slopes in similar ways regardless of whether individuals were in the intervention or control group.

#### 3.4.2. Teen-Reported Monitoring

For teen-reported parental monitoring, the model for the control group differed from the model for the intervention group in several ways. Although income was significantly associated with increased monitoring at baseline and teen girls reported marginally more monitoring than teen boys at baseline, neither parents' beliefs about adolescent risk-behavior being normative nor parents' trust and privacy beliefs were associated with parental monitoring slopes. Thus, parenting beliefs were differentially associated with patterns of monitoring over time based on whether teens' families were in the intervention or control groups.

## 4. Discussion

 Family interventions are often designed to alter family dynamics and parenting behaviors. Changes in parenting practices are then hypothesized to directly lead to reductions in adolescent problem behavior, such as risky sexual activity. However, research on the effects of parenting on children and adolescent developmental and behavioral outcomes has consistently demonstrated between-family heterogeneity in parenting beliefs, style, and behaviors [25]. Little research has considered ways in which these existing family and parent characteristics, such as parental attitudes, may interact with dimensions of family interventions. The current study explored associations between parent attitudes (concerning monitoring, adolescent risk taking, and teen privacy) and parent open communication and direct monitoring strategies in a sample of families enrolled in the COPA parental monitoring intervention and in a comparison control group. Findings indicated that parent attitudes concerning teen delinquency and risk taking being normative and beliefs in the importance of trusting and respecting their teens' privacy were longitudinally associated with change in parental monitoring strategies among families in the intervention group, but not in the control group. In contrast, parental attitudes were similarly related to longitudinal patterns of open communication in both the intervention and the control groups.

### 4.1. Open Communication

 Models examining associations between baseline parental attitudes and parent-reported open communication were nearly identical for the intervention and the control groups. In both models, parents who had positive attitudes toward the efficacy of monitoring reported higher levels of open communication at baseline assessment. Both high-quality communication and high levels of parental knowledge have been associated with positive parent-adolescent relationships [26], and parents who view direct monitoring practices as efficacious may engage in more conversations with their teens in which they seek to gain information about their teens' whereabouts. Increased parent-adolescent conversations may lead parents to view their relationship with their teens as entailing high levels of open communication. In contrast, parents who expressed beliefs regarding the normative nature of adolescent delinquency and risk behavior reported lower levels of open communication at baseline. Parents who believe risk taking is an inevitable part of being a teenager may see little utility in directly engaging in conversations with their child, leading them to report lower levels of open communication.

 Parental attitudes entailing high levels of trust and respect for teens' privacy were positively associated with increasing open communication across the four measurement waves of the study. Research has indicated that quality parent-adolescent communication diminishes across adolescence [27]. However, parents who do not put a premium on respecting their child's privacy may engage in secretive or indirect monitoring behaviors. Adolescents view such parental behaviors as invasion of their privacy which is associated with increased parent-adolescent conflict and reduced adolescent disclosure [28]. In contrast, parents who trust their teens and respect their privacy are more likely to engage in autonomy-supportive behaviors [29], which may, in turn, improve adolescent trust and increase opportunities for quality parent-adolescent communication.

 The above findings were similar in the control and intervention groups. The COPA intervention had been designed to specifically enhance monitoring-related behaviors, and we had hypothesized that open communication would be related to parental monitoring. However, open communication was not significantly correlated with teen-reported parental monitoring, so it might not be surprising that the models indicated that parenting attitudes did not interact with the intervention in predicting open communication. Instead, findings potentially point to normative, developmental processes whereby parent attitudes affect subsequent communication between parents and adolescents. Open communication may be central to other family-based interventions, especially those designed to enhance parent-adolescent discussion of sexual risk behavior and AIDS. Parental attitudes toward teens' sexually-specific risk behavior and beliefs concerning teens' right to privacy regarding their sexual lives may impact the effectiveness of such interventions. It may be imperative that future interventions account for these existing parental attitudes, as the current study indicates that such parental conceptualizations impact levels of open communication.

### 4.2. Parental Monitoring

 In contrast to models predicting patterns of open communication, associations between parental attitudes, and teen-reported parental monitoring differed between intervention and control group models. Within the intervention group model but not the control group model, parental beliefs about the normative nature of teen risk taking behavior were positively associated with longitudinal change in parental monitoring. When parents believed that teenage risk-taking was normal and to be expected, adolescents reported increasing parental monitoring across the four waves of the study. However, this finding was qualified by a quadratic effect (combined effects displayed in Figure 3). When parents viewed risk behavior as normative, teens initially reported a sharp increase in parental monitoring from baseline to 4-month assessment, but reported gradually less monitoring over the final two waves of the study. The instruction in the intervention combined with established attitudes that problem behavior was normative may have led these parents to escalate their monitoring behaviors right after the intervention. However, parents' belief that problem behavior was unavoidable potentially led them to see little utility in maintaining high levels of supervision. Research has found that parents' beliefs concerning the legitimacy of parental authority are associated with their engagement in direct monitoring behaviors such as solicitation [26]. Interestingly, parents who did not believe teen delinquency was normative initially reduced their monitoring behaviors, but then gradually increased their levels of monitoring across the remaining waves. For these parents, the effects of the intervention on their monitoring behaviors may have been slightly delayed due to their established beliefs about normal adolescent development. It is important to note that similar, though nonsignificant, coefficients were found in the control group model, so it is likely that the intervention intensified a more normative association between parenting attitudes and monitoring behaviors.

 In contrast, the effects of parenting beliefs regarding trust and privacy on the teen-reported monitoring time slopes were greatly disparate across the control and intervention group models. Interestingly, while teen-reported monitoring decreased across time in the intervention group, parents' positive attitudes toward privacy and trust exacerbated this downward trend. The COPA intervention encouraged parents to actively solicit information from their teens and engage in other direct monitoring activities. However, parents who viewed their teenagers as trustworthy and believed it was important to respect teens' privacy may have considered such parenting behaviors to be violations of their teens' trust leading to a reactive stance and engagement in fewer monitoring activities over time. It is important to note that trust/privacy beliefs were not associated with differences in monitoring behavior at baseline assessment. Thus, it is possible that the intervention was initially effective at increasing these parents' monitoring activities to a level similar to parents who expressed lower trust/privacy beliefs. However, parents' attitudes interfered with the long-term maintenance of the monitoring behaviors learned in the intervention. An alternative explanation is that parents who had higher levels of trust in their teens encouraged more frequent disclosure on the part of the adolescent [30], which reduced the need for them to engage in active monitoring strategies.

### 4.3. Application to Behavioral Interventions

 The current study demonstrates that parents vary in their attitudes concerning normative adolescent development and the efficacy of monitoring activities. The attitudes of parents enrolled in the intervention predicted longitudinal change in their monitoring activities, indicating that parental beliefs influenced whether the intervention was successful in establishing long-term changes in parenting activities aimed at reducing teen problem behavior. One explanation for this pattern of findings is that parents' who hold different beliefs may be more or less open to receiving instruction. Parents who see little utility in increasing their monitoring activity either because they view delinquency as inevitable or because they view such behavior as potentially disruptive to their relationship with their teen may be less likely to invest themselves wholeheartedly in the didactic components of an instructional intervention. Even if they participate fully, long-held beliefs may interfere with parents' willingness to consistently apply what they have learned, especially over an extended period of time. Interestingly, it was parents' attitudes about trust/privacy and delinquency and not their attitudes about the efficacy of monitoring which were associated with patterns of change in parental monitoring. Thus, it is important to consider not only individual's attitudes toward the specific goals of the intervention, but also related beliefs which may affect individual's views of the intervention.

 Findings from this study demonstrate the importance of considering ways in which individuals' beliefs and attitudes may influence receptivity to an intervention aimed at decreasing risky behavior. The current study examined heterogeneity regarding relatively innocuous parental attitudes. However, parents may have more strongly-held beliefs about more sensitive topics such as attitudes toward sexual behavior beliefs about their children being at risk for sexually transmitted infections such as AIDS. The effectiveness of an intervention aimed at increasing parent-adolescent communication about such potentially “touchy” issues may be heavily influenced by parents' attitudes. Interventions which are not sensitive to individual beliefs or flexible enough to account for variability in attitudes may be ineffective at changing behavior.

### 4.4. Limitations and Future Directions

 The current study has multiple methodological strengths including the use of short-interval (4 months) longitudinal data and the use of multi-informant data (parents and teens). However, these strengths and the findings should be interpreted in light of the studies' limitations. First, the final sample enrolled for the randomized controlled trial of the COPA intervention included unequal representation of boys and girls. The greater proportion of girls and mothers participating in the study limit the application of these finding to fathers and sons. The study samples were also recruited from a rural setting and, therefore, do not represent the same parental attitudes found within families of suburban or inner city areas.

 With an approach that examined new parent attitudes about monitoring, this study expands upon our understanding of the different ways in which parent-adolescent dyads might respond to a parental monitoring intervention program. Understanding the way in which parents' monitoring attitudes might affect their interactions with their adolescents and particular choices of monitoring will be needed in future studies. Based on these initial findings, a greater assessment of parent monitoring attitudes, and would be needed to identify additional program strategies for enhancing parent-adolescent communication and direct monitoring. More generally, the current study argues for the importance of considering heterogeneity in individual attitudes and beliefs when designing and implementing behavioral interventions.

## Figures and Tables

**Table 1:** Study sample characteristics valid percentages, and mean reporting.

Characteristic	Intervention group (*N* = 339)				Control group (*N* = 173)			
	*N*	(%)	*M* (SE)	SD	*N*	(%)	*M* (SE)	SD
Gender (female)	251	74.0			108	62.4		
Race (Caucasian)	323	95.6			167	96.5		
Two/less children in home	229	85.4			119	83.8		
Stepchildren in the home	19	5.7			12	7.1		
Two adults in the home	250	74.0			135	78.0		
Family income ≤15,000	64	19.2			32	18.9		
Attitudes—UMP			3.5 (0.02)	0.33			3.5 (0.02)	0.34
Attitudes—EXP			2.6 (0.02)	0.38			2.6 (0.03)	0.35
Attitudes—ATP			1.7 (0.02)	0.54			1.7 (0.04)	0.57
Parent-reported open communication—baseline			3.2 (0.02)	0.40			3.2 (0.03)	0.41
Parent-reported open communication—4 month			3.1 (0.02)	0.36			3.1 (0.04)	0.37
Parent-reported open communication—8 month			3.1 (0.03)	0.38			3.1 (0.04)	0.37
Parent-reported open communication—12 month			3.1 (0.02)	0.39			3.2 (0.04)	0.42
Adolescent-reported direct monitoring—baseline			2.8 (0.05)	0.96			2.8 (0.07)	0.94
Adolescent-reported direct monitoring—4 month			2.8 (0.07)	1.00			2.8 (0.09)	0.98
Adolescent-reported direct monitoring—8 month			2.6 (0.07)	0.98			2.7 (0.09)	0.96
Adolescent-reported direct monitoring—12 month			2.7 (0.08)	1.02			2.7 (0.10)	0.97

UMP: parent attitudes about the usefulness of the monitoring process; EXP: parent attitudes about the impact of monitoring on adolescent risk behavior and experimentation; ATP: parent attitudes about monitoring and the importance of adolescent trust and privacy.

**Table 2:** Pearson correlations among study variables (intervention participants, *N* = 339).

	1	2	3	4	5	6	7	8	9	10
(1) UMP	—									
(2) EXP	−.042	—								
(3) ATP	−.445**	.064	—							
(4) Parent-reported open communication—baseline	.494**	−.271**	−.244**	—						
(5) Parent-reported open communication—4 month	.306**	−.183**	−.117	.643**	—					
(6) Parent-reported open communication—8 month	.291**	−.126	−.080	.477**	.534**	—				
(7) Parent-reported open communication—12 month	.340**	−.228**	−.046	.565**	.632**	.589**	—			
(8) Adolescent-reported direct monitoring—baseline	.091	−.046	−.090	.080	.029	−.005	.059	—		
(9) Adolescent-reported direct monitoring—4 month	.028	.022	−.122	−.004	−.030	.005	−.138	.629**	—	
(10) Adolescent-reported direct monitoring—8 month	.097	.027	−.169*	.075	.013	.022	−.033	.561**	.679**	—
(11) Adolescent-reported direct monitoring—12 month	.127	−.080	−.202**	.131	.075	.060	.055	.530**	.573**	.623**

* = *P* < .05; **: *P* < .01; UMP: parent attitudes about the usefulness of the monitoring process; EXP: parent attitudes about the impact of monitoring on adolescent risk behavior and experimentation; ATP: parent attitudes about monitoring and the importance of adolescent trust and privacy.

**Table 3:** Hierarchical linear growth model for parent-reported open communication.

	Intervention group			Control group		
Variable	Coefficient	SE	*P* value	Coefficient	SE	*P* value
Intercept	2.97			1.97		
Income	−.03	.012	.033	−.01	.018	.607
Teen age	.02	.012	.050	−.02	.019	.208
Parent gender (female)	.16	.056	.005	−.07	.088	.425
UMP	.53	.052	<.001	.53	.072	<.001
EXP	−.23	.041	<.001	−.32	.065	<.001
Linear slope (wave)	−.02	.008	.03	.003	.012	.809
UMP	−.05	.027	.06	.01	.038	.72
ATP	.04	.015	.018	.05	.022	.022

UMP: parent attitudes about the usefulness of the monitoring process; EXP: parent attitudes about the impact of monitoring on adolescent risk behavior and experimentation; ATP: parent attitudes about monitoring and the importance of adolescent trust and privacy.

**Table 4:** Hierarchical linear growth model for teen-reported parental monitoring.

	Intervention group			Control group		
Variable	Coefficient	SE	*P* value	Coefficient	SE	*P* value
Intercept	2.53			2.71		
Income	.15	.037	<.001	.14	.048	.004
Teen gender (female)	.35	.103	<.001	.24	.126	.064
Linear slope (wave)	−.11	.068	.096	−.02	.101	.819
EXP	.40	.169	.017	.31	.251	.220
ATP	−.10	.034	.007	.07	.057	.219
Quadratic slope (wave)	.03	.023	.198	−.01	.033	.734
EXP	−.14	.058	.021	−.09	.087	.306

UMP: parent attitudes about the usefulness of the monitoring process; EXP: parent attitudes about the impact of monitoring on adolescent risk behavior and experimentation; ATP: parent attitudes about monitoring and the importance of adolescent trust and privacy.
